# Supplementary material for: Polymer-constrained excimer enables flexible and self-healable optoelectronic elastomer for mechanical sensor
Source: Nat Commun. 2025 Nov 25;16:10500. doi: 10.1038/s41467-025-65539-9 (PMC12647883; doi:10.1038/s41467-025-65539-9)
Supplement: Supplementary file 1 — Supplementary Information [file 41467_2025_65539_MOESM1_ESM.pdf]

Supplementary Information

Polymer-Constrained Excimer Enables Flexible and Self-Healable Optoelectronic Materials for Wearable Sensor

Shuyu Zheng,[1,2] Dazhe Zhao,[3] Nengjie Cao,[1] Jiajia Zhou,[1,2,*] Junwen Zhong, [3,*] Haobing Wang [1,2,*]

Author affiliations:

1 Advanced Institute for Soft Matter Science and Technology (AISMST), School of Emergent Soft Matter, South China University of Technology 510640 Guangzhou, China

2 Guangdong Provincial Key Laboratory of Functional and Intelligent Hybrid Materials and Devices, Guangdong Basic Research Center of Excellence for Energy and Information Polymer Materials, South China University of Technology, 510640 Guangzhou, China

3 Department of Electromechanical Engineering and Centre for Artificial Intelligence and Robotics, University of Macau, Macau, SAR 999078, China

*Correspondence to: junwenzhong@um.edu.mo (J. Zhong), zhouj2@scut.edu.cn (J. Zhou), haobingwang@scut.edu.cn (H. Wang)

Supplementary Table of the content

1. Supplementary Methods

Characterizations

The calculation of reactivity ratio

The calculation of photoluminescence quantum yield

2. Supplementary Figures and Tables

Supplementary Fig. 1. Reaction processes characterization of PNI copolymer. ^{1}H-NMR spectrum (400 MHz, $CDCl_3$) of poly(1-vinylnaphthalene-*co*-isoprene) copolymer (PNI), isoprene (IP), and 1-vinylnaphthalene (VN).

Supplementary Fig. 2. Reaction processes characterization of IP and styrene copolymer. ^{1}H NMR spectra of tracing the copolymerization of IP with styrene (St).

Supplementary Table 1. Preparation of copolymer. Copolymerization of isoprene (IP) and 1-vinylnaphthalene (VN).

Supplementary Fig. 3. ^{1}H-NMR spectrum of PNI copolymer and PVN. ^{1}H-NMR spectrum (400 MHz, $CDCl_3$) of poly(1-vinylnaphthalene) (PVN) and poly(1-vinylnaphthalene-co-isoprene) copolymer (PNI) with different contents of 1-vinylnaphthalene (VN): 10, 20, 25, 30, 35, 40, 50, and 70 mol%.

Supplementary Fig. 4. GPC curve of PNI copolymer. GPC curve of **a**) PNI copolymer (25 ± 5 kDa) with different content of VN (10, 20, 30, 40, 50, and

70 mol%), and **b**) PNI copolymer (27-28 mol% VN) with different molecular weight (117, 143, 184, 201, and 227 kDa).

Supplementary Fig. 5. ^{1}H DOSY NMR spectrum of P1. ^{1}H DOSY NMR spectrum (400 MHz, $CDCl_3$) with 1D spectrum shown at the top of P1.

Supplementary Fig. 6. NMR test of copolymer. ^{13}C-NMR spectrum (100 MHz, $CDCl_3$) of P3.

Supplementary Fig. 7. Thermogravimetric Analysis (TGA) of PNI copolymers. Thermo Gravimetric Analysis (TGA) of PNI copolymer with different contents of VN: 10, 20, 25, 30, 35, 40, 50, and 70 mol%.

Supplementary Table 2. Mechanical test of copolymer. Stress-strain test of PNI copolymers.

Supplementary Fig. 8. Mechanical test of copolymer. Cyclic stress-strain test for P3.

Supplementary Fig. 9. Self-healing test of P5 and PNI with 35 mol% VN. **a**) Self-healing test of P5. **b**) Self-healing test of PNI copolymer (180 kDa, 35 mol% VN). **c**) Self-healing comparison of P3 self-healed under room temperature and human body temperature, and P5 self-healed under room temperature.

Supplementary Fig. 10. Dynamic mechanical analysis of PNI copolymer. Dynamic mechanical analysis of **a**) PNI copolymer with different contents of VN (10, 30, and 50 mol%), and **b**) PNI copolymer of 27 mol% VN with different molecular weight (117, 184, and 227 kDa).

Supplementary Fig. 11. Dynamic mechanical analysis of PNI copolymer and segmented copolymer. Dynamic mechanical analysis of PNI copolymer with 184 kDa and 27 mol% VN (top) and segmented copolymer of NIN-3 (bottom, 22 kDa, 30 mol% VN).

Supplementary Fig. 12. Rheological measurement curves of PNI copolymer. Rheological measurement curves: Storage modulus of PNI copolymer (M_n = 24 ± 4 kDa) with **a**) 10 mol% VN, **b**) 30 mol% VN, and **c**) 50 mol% VN. Loss modulus of PNI copolymer with **d**) 10 mol% VN, **e**) 30 mol% VN, and **f**) 50 mol% VN.

Supplementary Fig. 13. Rheological measurement curves of PNI copolymer. Rheological measurement curves: Storage modulus of PNI copolymer (27 mol% VN) with M_n = **a**) 117 kDa, **b**) 184 kDa, and **c**) 227 kDa. Loss modulus of PNI copolymer (27 mol% VN) with M_n = **d**) 117 kDa, **e**) 184 kDa, and **f**) 227 kDa.

Supplementary Fig. 14. Rheological measurement curves of PNI copolymer and segmented copolymer. Rheological measurement curves: Storage modulus of **a**) P3, **b**) segmented copolymer of NIN-3. Loss modulus of **c**) P3, **d**) NIN-3.

Supplementary Fig. 15. Schematic diagram of the test principle. Measurement of quantum yield of P3 film using the integrating sphere.

Supplementary Fig. 16. Fluorescence test of copolymer. The fluorescence intensity variation of P3 during self-healing process.

Supplementary Fig. 17. Fluorescent spectra of Naphthalene and PNI copolymer dissolved in THF. **a**) Naphthalene dissolved in THF (1 mM). **b**) P3 dispersed in THF (18 g/L).

Supplementary Fig. 18. Fluorescence test of copolymer. Fluorescent emission spectra of PNI copolymer with different content of VN (10, 20, 25, 30, 35, 40, and 50 mol%).

Supplementary Fig. 19. Fluorescent confocal test of copolymer. The 3D reconstruction of P3 block by Z-stack scan of the fluorescent confocal microscope.

Supplementary Fig. 20. Scanning electron microscope (SEM) images and BET test result of P3. **a**) and **b**) Scanning electron microscope (SEM) images of P3. **c**) Pore size distribution curve of P3 gained by Brunauer-Emmett-Teller test (BET Surface Area: 0.4417 m^2/g).

1. Supplementary Methods

Characterizations. Nuclear magnetic resonance (NMR) spectra were recorded on a 400/100 MHz (Bruker, Fällanden, Switzerland) (FT, 400 MHz for ^{1}H; 100 MHz for ^{13}C) spectrometer with $CDCl_3$ as solvent. Chemical shifts were referenced to the signal of the solvent (residual proton resonances for ^{1}H spectra, carbon resonances for ^{13}C spectra).

Molecular weights and molecular-weight distributions of the polymers were determined by gel permeation chromatography (GPC). Thermal gravimetric analysis (TGA) on a Mettler Toledo TGA/DSC 3+ STARe System, TA Instrument, was used to determine the decomposition temperature of polymers; the samples were heated from ambient temperature to 900 °C at a rate of 10 °C min^{-1}.

Differential scanning calorimetry (DSC) measurements were performed on a DSC (Mettler Toledo DSC 3). Polymer samples were weighed (typically in a 3-5 mg range) and sealed in hermetic aluminum pans using a DSC press. The samples were first heated to 250 °C at 10 °C min^{-1}, equilibrated at this temperature for 5 min, then cooled to −60 °C at 10 °C min^{-1}, held at this temperature for 5 min, and reheated to 250 °C at 10 °C min^{-1} under a nitrogen flow (50 mL/min). All T_g values were obtained from the second scan.

The viscoelastic properties of polymers were analyzed using a Discovery DMA 850. The testing was made in compression mode at 200

mN with heating from −75 to 240 °C at a heating rate of 10 °C min^{-1} and a fixed frequency of 1 Hz. Rheological measurements were carried out by Anton Paar 302e, Austria. The samples were tested from −30 to 150 °C, with an angular frequency from 0.1 to 100 rad/s.

Mechanical tensile-stress experiments were performed using an Instron 68SC-1 instrument. Three samples were tested for each polymer composition. Tensile experiments were performed at room temperature (25 ± 1 °C) at different sample size and strain rate when evaluating the stretchability according to ASTM 882-09 test method using dumbbell-conFig.d specimens according to JIS K-6251-7 (width: 2 mm; length: 12 mm; thickness: 1 mm). Strain at break and stress at break experiment was determined at fracture using uniaxial tensile test with a strain rate of 300 mm/min. Young's modulus is the initial slope of the nominal stress vs nominal strain curve in the linear region ($0 < \varepsilon < 0.05$) and was calculated from the average of three monotonic curves. Cyclic stress-strain test for P3 was carried out with a strain rate of 300 mm/min and release rate of 300 mm/min. Strain recovery was determined by a 550 % strain step cycle test using the equation $100\ (\varepsilon_a - \varepsilon_r)/\varepsilon_a$, where ε_a = applied strain and ε_r = strain at zero load after 10th cycle.

For self-healing tests, the sample was cut into separate parts completely using razor blade. The break surface of films was contacted in air. The cut faces were brought together and gently pressed for less than 15

seconds at 25 °C. The healed polymer films were then stretched following the same procedure to obtain the stress-strain curves. The mechanical healing efficiency was defined as the ratio between the fracture strain restored relative to the original fracture strain.

Transmission Electron Microscopy (TEM) images were taken by Talos F200X. Visible light transmittance spectrum was taken by SHIMADZU UV-3600 PLUS, the sample is made by hot-pressing with a thickness of 50 μm.

Fluorescent (FL) excitation and emission spectra were taken by HITACHI F-4700. The liquid samples were tested by using quartz cell, and the solid samples were tested by being fixed on a professional fixture. The quantum yields of the samples were taken by Quantaurus-QY Plus C13534-11, Hamamatsu Photonics, and calculated by the bundled software. Fluorescent optical images were taken by one of two confocal laser scanning microscopy (CLSM) systems, Leica TCS SP8 STED or Zeiss SLM 880. Scanning electron microscope (SEM) images were taken by SIGA 300.

Brunauer-Emmett-Teller test was taken by Micromeritics ASAP 2460. The sample is made by hot-pressing and cut into small pieces, then the sample was put into the glass container and dried at reduced pressure under 80 °C by Micromeritics VacPrep 061 Sample Degas System over night and used for the following test.

The corona charging of the PNI copolymer film was taken by Dongwen DW-P303-1ACD1 (High Voltage Power Supply). The surface potential of the samples is measured by an electrostatic voltmeter (Trek 347). The output current and voltage of the samples are measured by a Keithely 6514 electrometer and an NI USB 6341 data acquisition system.

The calculation of reactivity ratio. The reactivity ratios of IP and VN were calculated by linear fitting, and the raw data were taken from ^{1}H NMR spectra of PNI copolymerization with different feed ratio. The reactivity ratio of IP and VN can be expressed by equations as follows:

$$\frac{d[M_1]}{d[M_2]} = \frac{[M_1]}{[M_2]} \cdot \frac{r_1[M_1]+[M_2]}{r_2[M_2]+[M_1]} \tag{1}$$

where $d[M_1]$ and $d[M_2]$ are respectively the substance amount of M_1 and M_2 in the copolymer chain at a certain moment, $[M_1]$ and $[M_2]$ are respectively the substance amount of monomer M_1 and M_2, r_1 and r_2 are respectively the reactivity ratio of monomer M_1 and M_2.

$$R = \frac{[M_1]}{[M_2]} \tag{2}$$

where R is the initial molar ratio of the two monomers.

$$\rho = \frac{d[M_1]}{d[M_2]} \tag{3}$$

where ρ is the molar ratio of the two monomer units in the copolymer produced at this instant.

According to equation (1), (2), and (3), the equation (1) can be expressed as follows:

$$\frac{R(\rho-1)}{\rho} = \frac{R^2}{\rho} r_1 - r_2 \tag{4}$$

The calculated result is shown in Supplementary Fig. 2c.

The calculation of photoluminescence quantum yield. The IUPAC definition of (integral) quantum yield, $\Phi(\lambda)$ is [1]:

$$\Phi\ (\lambda) = \frac{\text{number of events}}{\text{number of photons absorbed}} \tag{5}$$

where $\Phi\ (\lambda)$ can be used for photophysical processes. More usually, it is written for emission of light:

$$\Phi\ (\lambda) = \frac{\text{number of photons emitted}}{\text{number of photons absorbed}} \tag{6}$$

To determine the PLQY Φ, we use the de Mello method with three measurements, as shown in Supplementary Fig. 15. First, the excitation is led into the empty sphere to quantify the excitation intensity (empty sphere, coded as A). Second, the sample is placed within the sphere, but not in the excitation beam (indirect illumination of the sample, coded as B). Third, the sample has to be placed directly in the excitation beam (direct illumination, coded as C). For a wavelength scale, the integration in each case should be with respect to $\int \frac{\lambda}{hc} d\lambda$, and each spectrum taken consists of two parts: the residual excitation light and the emitted light. Both parts are integrated separately and are referred to as X (excitation) and E (emission), with a subscript denoting the respective measurement. With this, the absorption A and finally the PLQY Φ can be calculated [2, 3, 4]:

$$A = (1 - \frac{X_\mathrm{C}}{X_\mathrm{B}}) \tag{7}$$

$$\Phi = \frac{E_\mathrm{C} - (1-A)E_\mathrm{B}}{A \cdot X_\mathrm{A}} \tag{8}$$

2. Supplementary Figures and Tables

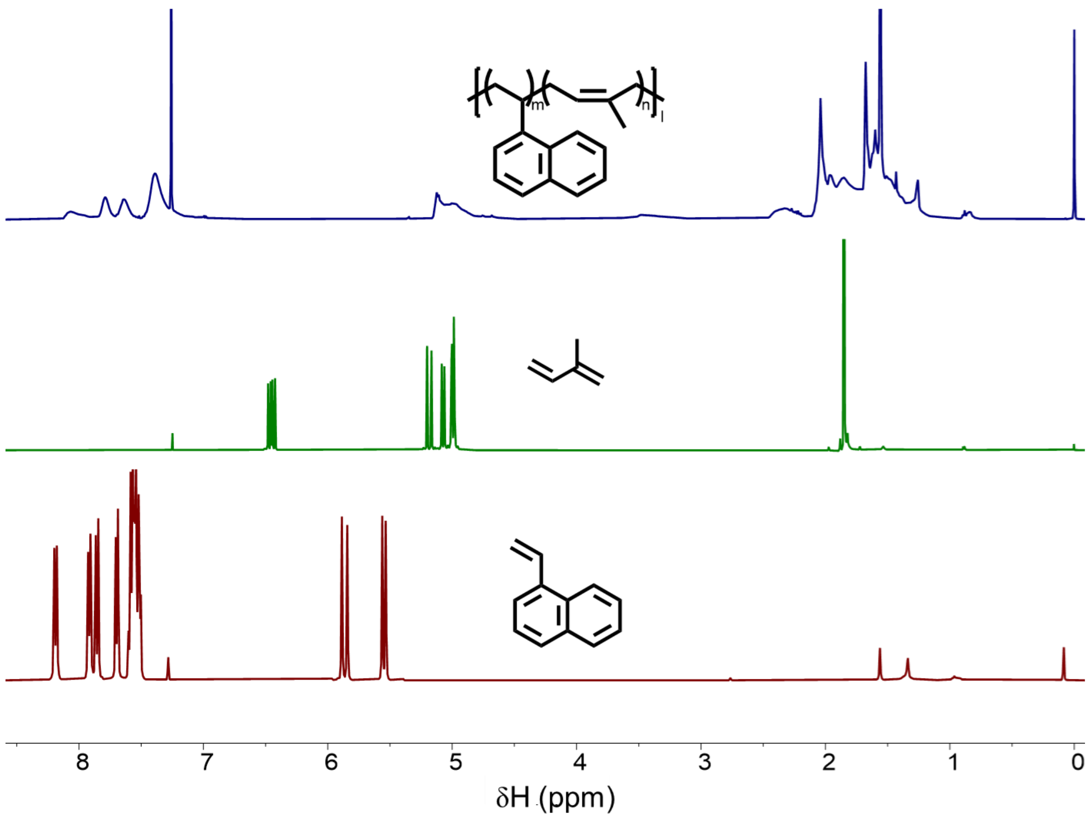

Supplementary Fig. 1. Reaction processes characterization of PNI copolymer. ^{1}H-NMR spectrum (400 MHz, $CDCl_3$) of poly(1-vinylnaphthalene-*co*-isoprene) copolymer (PNI), isoprene (IP), and 1-vinylnaphthalene (VN).

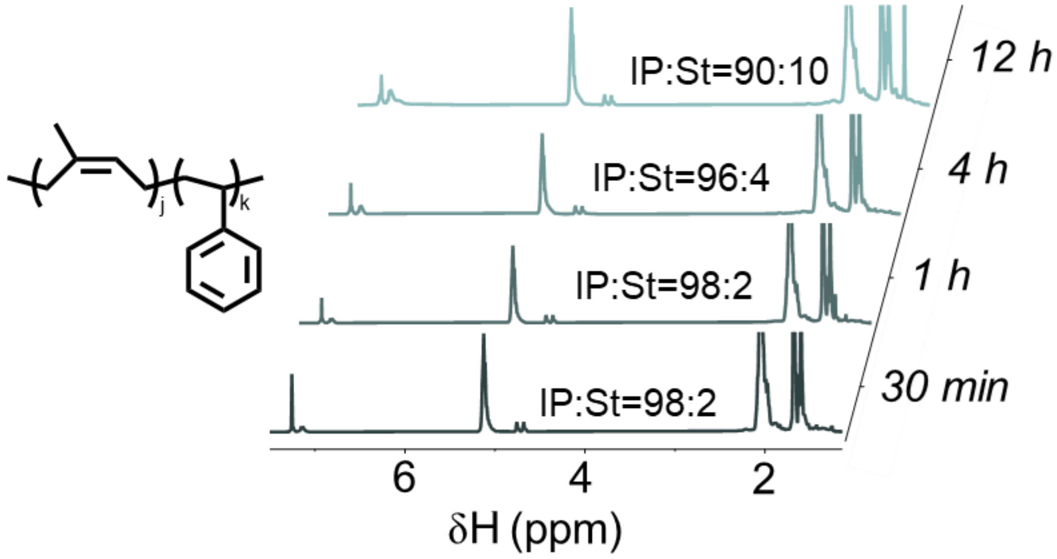

Supplementary Fig. 2. Reaction processes characterization of IP and styrene copolymer. ^{1}H NMR spectra of tracing the copolymerization of IP with styrene (St), (400 MHz, $CDCl_3$).

Supplementary Table 1. Preparation of copolymer. Copolymerization of isoprene (IP) and 1-vinylnaphthalene (VN).

Polymer type	[**IP**] : [**VN**] : [*s*-BuLi]^b	conv. **IP** (%)	conv. **VN** (%)	M_n (×10^3 g mol^{-1})^c	**IP**/**VN**^{d}	M_w/M_n^{c}	T_g (°C)^e
Random	310 : 40 : 1	95 %	81 %	28	90/10	1.1	−45
Random	280 : 70 : 1	90 %	85 %	27	80/20	1.2	−30
Random	240 : 110 : 1	95 %	89 %	25	70/30	1.2	5
Random	190 : 160 : 1	94 %	75 %	23	60/40	1.1	40
Random	160 : 190 : 1	84 %	70 %	21	50/50	1.1	70
Random	80 : 270 : 1	79 %	54 %	19	30/70	1.1	100
Homo	0 : 350 : 1	/	59 %	10	0/100	2.7	120
Random	1550 : 550 : 1	91 %	85 %	123	75/25	1.1	9
Random	1500 : 600 : 1	96 %	82 %	117	73/27	1.1	10
Random	1800 : 720 : 1	95 %	82 %	143	72/28	1.6	10
Random	2000 : 800 : 1	92 %	79 %	184	73/27	2.5	11
Random	2200 : 880 : 1	90 %	78 %	201	73/27	2.3	13
Random	2500 : 1000 : 1	90 %	77 %	227	73/27	2.3	15
Random	2500 : 300 : 1	90 %	83 %	201	90/10	1.5	−40
Random	1700 : 1100 : 1	89 %	74 %	180	65/35	3.5	20
Random	800 : 950 : 1	77 %	65 %	86	50/50	3.6	70
Segmented	240 : 110 : 1	83 %	78 %	22	70/30	1.5	90

a) Conditions: Sec-Butyllithium (*s*-BuLi) (1.3×10^{-2} mmol); 50 mL Cyclohexane; Room temperature. **b**) Feed ratio (in moles) of isoprene (IP), 1-vinylnaphthalene (VN), and *s*-BuLi. **c**) Determined by gel permeation chromatography (GPC) in tetrahydrofuran (THF) at 35 °C against polystyrene standard (Supplementary Fig. 4). M_n = number-average molecular weight, M_w = weight-average molecular weight. **d**) Molar ratio of IP and VN in the copolymer, determined by ^{1}H nuclear magnetic resonance (NMR) analysis. **e**) Determined by differential scanning calorimetry (DSC).

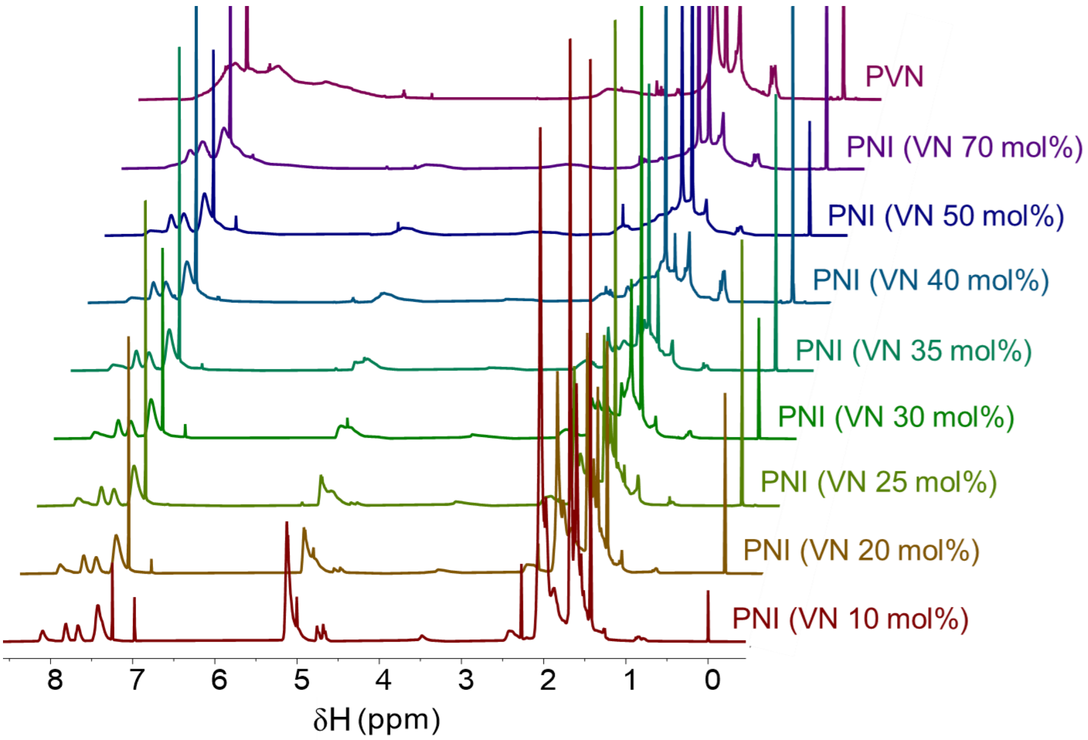

Supplementary Fig. 3. ^{1}H-NMR spectrum of PNI copolymer and PVN. ^{1}H-NMR spectrum (400 MHz, $CDCl_3$) of poly(1-vinylnaphthalene) (PVN) and poly(1-vinylnaphthalene-*co*-isoprene) copolymer (PNI) with different contents of 1-vinylnaphthalene (VN): 10, 20, 25, 30, 35, 40, 50, and 70 mol%.

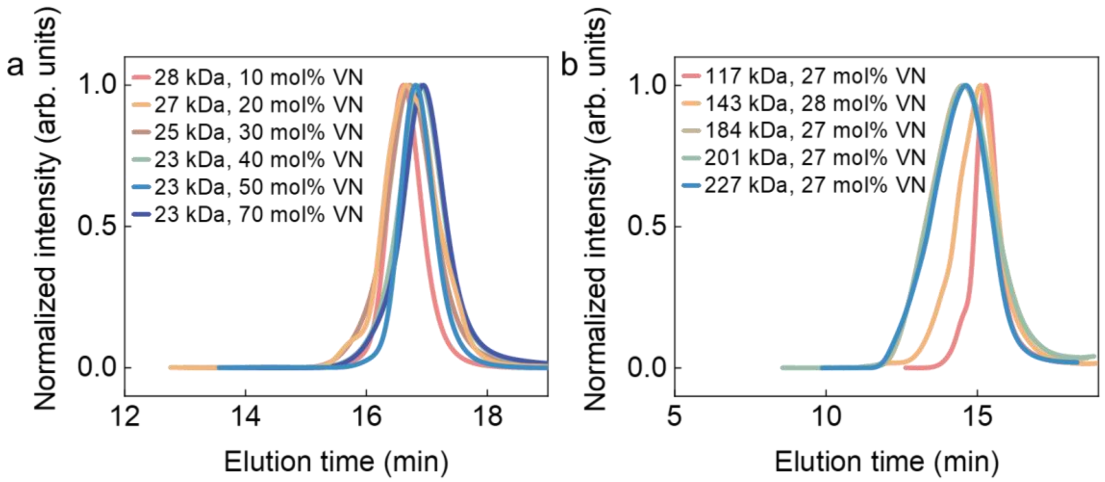

Supplementary Fig. 4. GPC curve of PNI copolymer. **a**) PNI copolymer (25 ± 5 kDa) with different content of VN (10, 20, 30, 40, 50, and 70 mol%), and **b**) PNI

copolymer (27-28 mol% VN) with different molecular weight (117, 143, 184, 201, and 227 kDa).

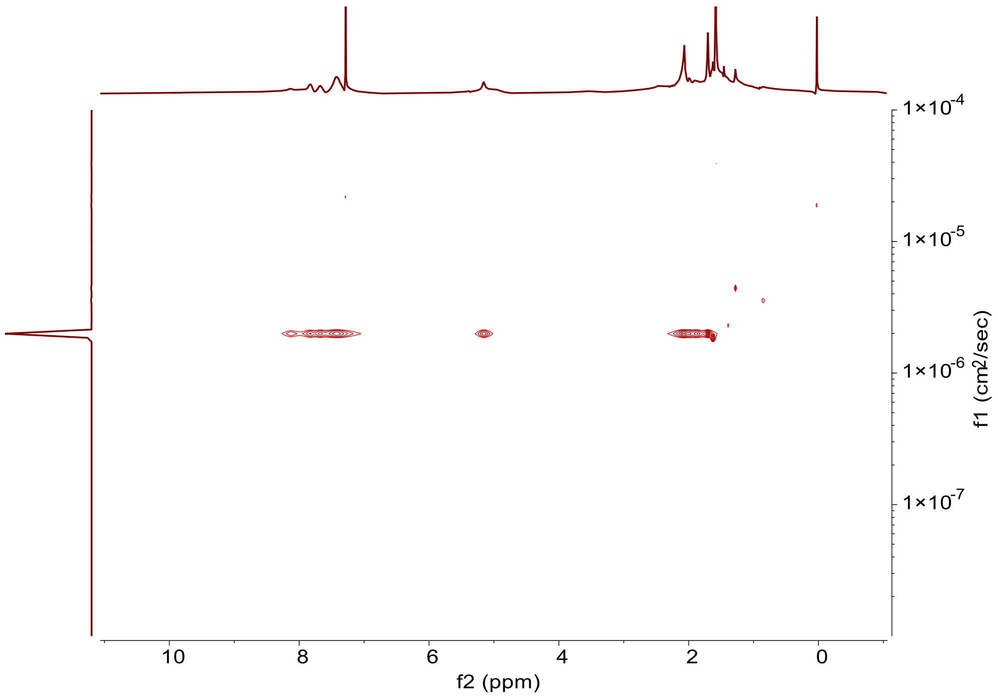

Supplementary Fig. 5. ^{1}H DOSY NMR spectrum of P1. ^{1}H DOSY NMR spectrum (400 MHz, $CDCl_3$) with 1D spectrum shown at the top of P1.

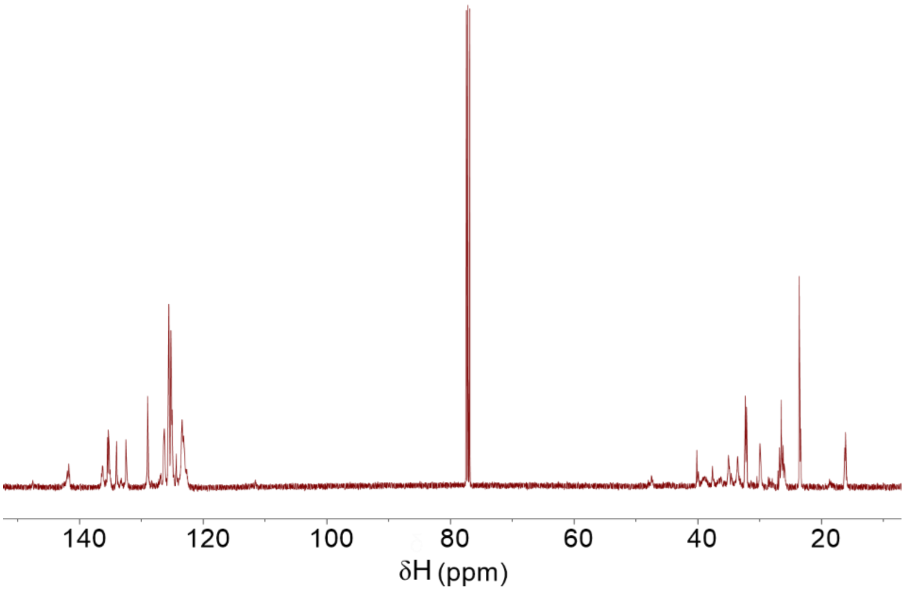

Supplementary Fig. 6. NMR test of copolymer. Typical ^{13}C-NMR spectrum (100 MHz, $CDCl_3$) of copolymer (P3).

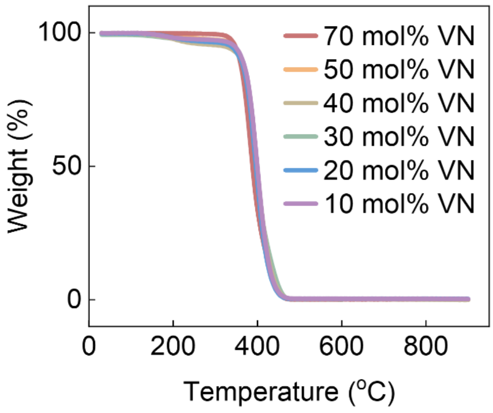

Supplementary Fig. 7. Thermogravimetric Analysis (TGA) of PNI copolymers. Thermogravimetric Analysis (TGA) of PNI copolymers with different VN content: 10, 20, 25, 30, 35, 40, 50, and 70 mol%.

Supplementary Table 2. Mechanical test of copolymer. Stress-strain test of PNI copolymers.

Content of VN (mol%) [a]	M_n ($\times10^3$ g mol^{-1}) [b]	breaking strength (MPa) [c]	elongation at break (%) [c]	Young's modulus (MPa) [c]	Toughness (MJ m^{-3}) [c]
10	28	0.14 ± 0.01	1460 ± 20	0.1 ± 0.05	2.3 ± 0.1
30	25	1 ± 0.1	2020 ± 20	0.5 ± 0.1	15 ± 1
40	23	25 ± 1	300 ± 10	100 ± 2	89 ± 2
50	21	24 ± 1	5 ± 1	460 ± 5	1.45 ± 0.05
70	19	13 ± 1	2 ± 1	400 ± 10	0.50 ± 0.05
27	117	2.4 ± 0.1	1950 ± 10	0.7 ± 0.1	31 ± 1
28	143	3 ± 0.5	1890 ± 5	5 ± 0.5	40 ± 1
27	184	12 ± 0.5	1450 ± 5	8 ± 1	95 ± 2
27	201	20 ± 1	1300 ± 5	13 ± 0.5	170 ± 3
27	227	25 ± 1	1200 ± 20	14 ± 1	180 ± 2

a) Molar ratio of 1-vinylnaphthalene (VN) in the copolymer, determined by ^{1}H nuclear magnetic resonance (NMR) analysis. b) Determined by gel permeation chromatography (GPC) in tetrahydrofuran (THF) at 35 °C against polystyrene standard (Supplementary

Fig. 4). M_n = number-average molecular weight. c) Determined by Stress-strain curves of PNI copolymer.

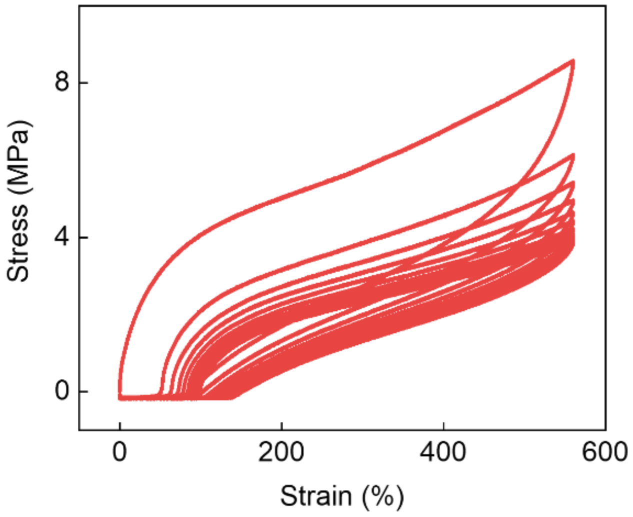

Supplementary Fig. 8. Mechanical test of copolymer. Cyclic stress-strain test for P3.

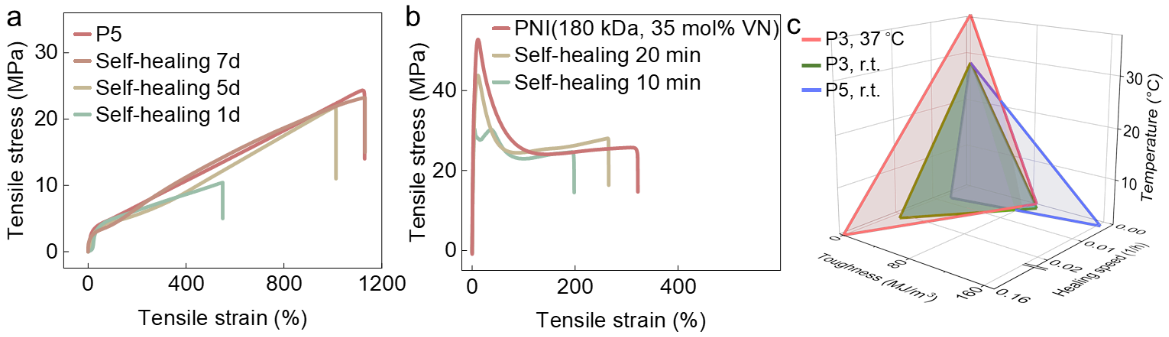

Supplementary Fig. 9. Self-healing test of P5 and PNI with 35 mol% VN. **a**) Self-healing test of P5. **b**) Self-healing test of PNI copolymer (180 kDa, 35 mol% VN). **c**) Self-healing comparison of P3 self-healed under room temperature and human body temperature, and P5 self-healed under room temperature.

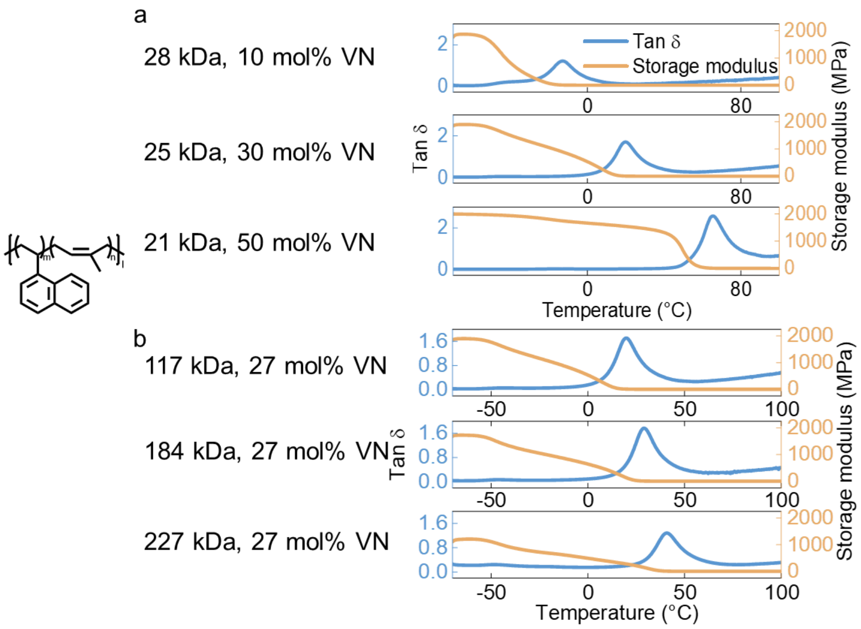

Supplementary Fig. 10. Dynamic mechanical analysis of PNI copolymer. Dynamic mechanical analysis of **a**) PNI copolymer with different contents of VN (10, 30, and 50 mol%), and **b**) PNI copolymer of 27 mol% VN with different molecular weight (117, 184, and 227 kDa).

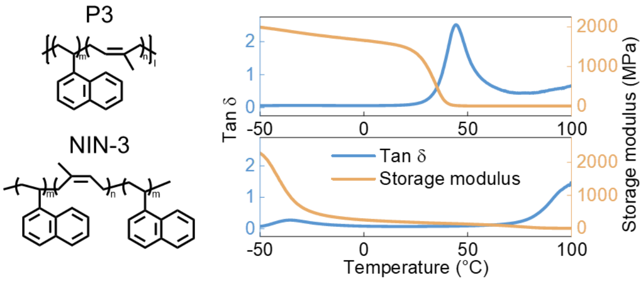

Supplementary Fig. 11. Dynamic mechanical analysis of PNI copolymer and segmented copolymer. Dynamic mechanical analysis of PNI copolymer with 184 kDa and 27 mol% VN (top) and segmented copolymer of NIN-3 (bottom, 22 kDa, 30 mol% VN).

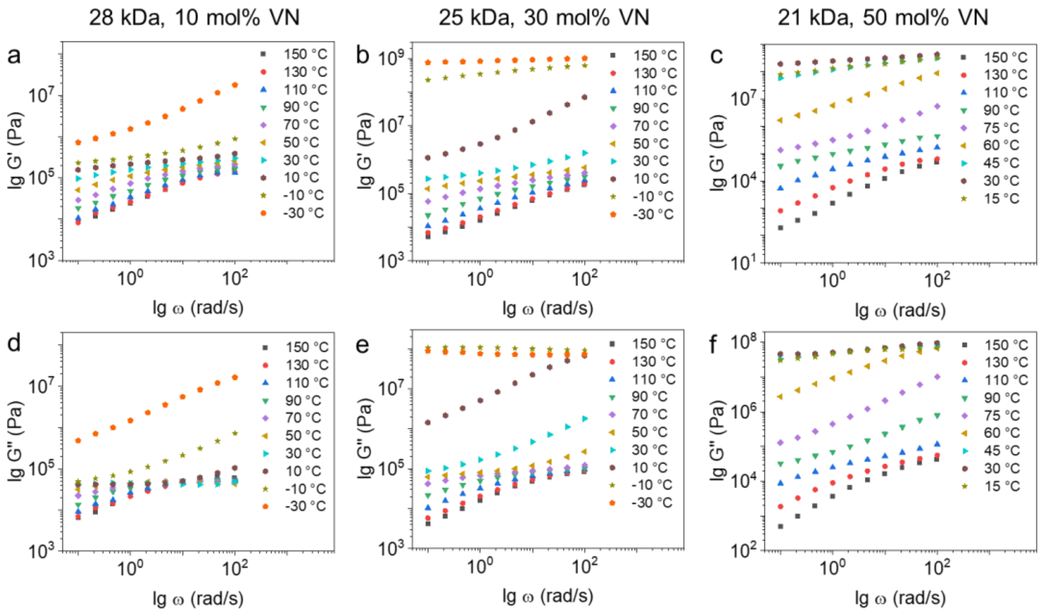

Supplementary Fig. 12. Rheological measurement curves of PNI copolymer. Rheological measurement curves: Storage modulus of PNI copolymer (M_n = 24 ± 4 kDa) with **a**) 10 mol% VN, **b**) 30 mol% VN, and **c**) 50 mol% VN. Loss modulus of PNI copolymer with **d**) 10 mol% VN, **e**) 30 mol% VN, and **f**) 50 mol% VN.

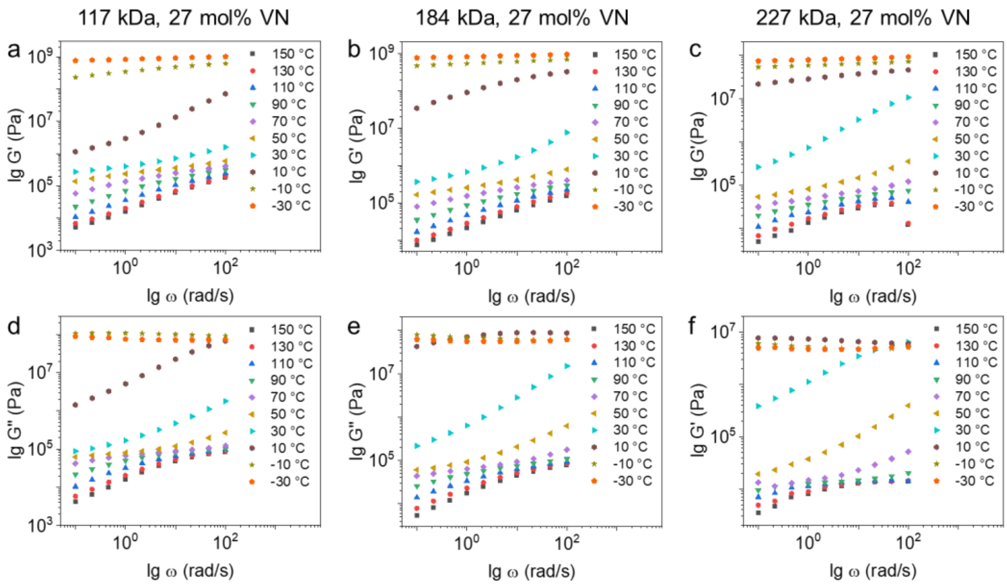

Supplementary Fig. 13. Rheological measurement curves of PNI copolymer. Rheological measurement curves: Storage modulus of PNI copolymer (27 mol% VN) with M_n = **a**) 117 kDa, **b**) 184 kDa, and **c**) 227 kDa. Loss modulus of PNI copolymer (27 mol% VN) with M_n = **d**) 117 kDa, **e**) 184 kDa, and **f**) 227 kDa.

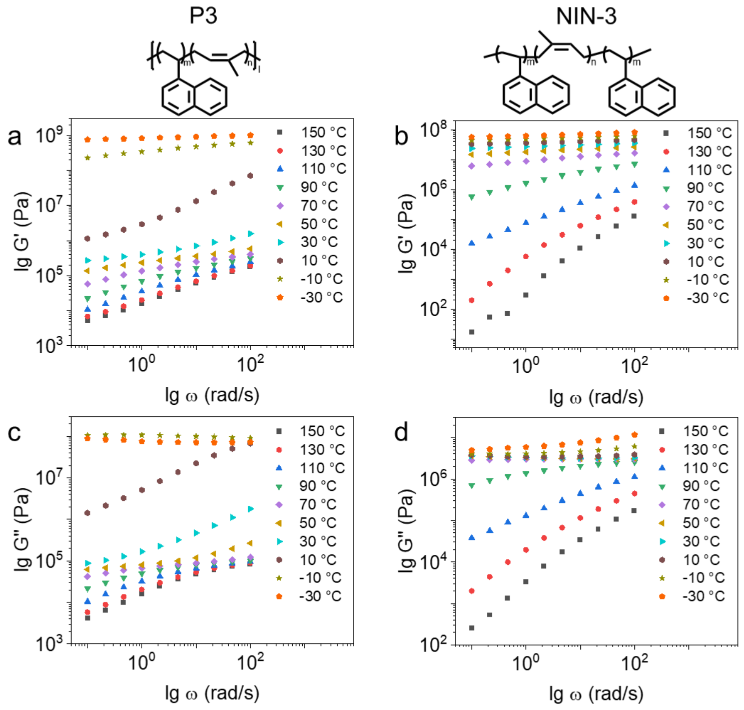

Supplementary Fig. 14. Rheological measurement curves of PNI copolymer and segmented copolymer. Rheological measurement curves: Storage modulus of **a**) P3, **b**) segmented copolymer of NIN-3. Loss modulus of **c**) P3, **d**) NIN-3.

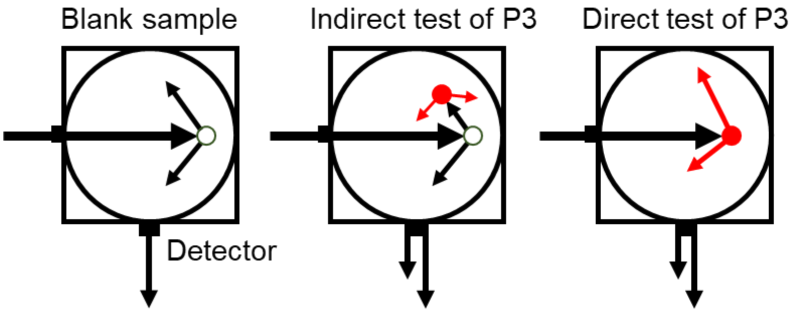

Supplementary Fig. 15. Schematic diagram of the test principle. Measurement of quantum yield of P3 film using the integrating sphere.

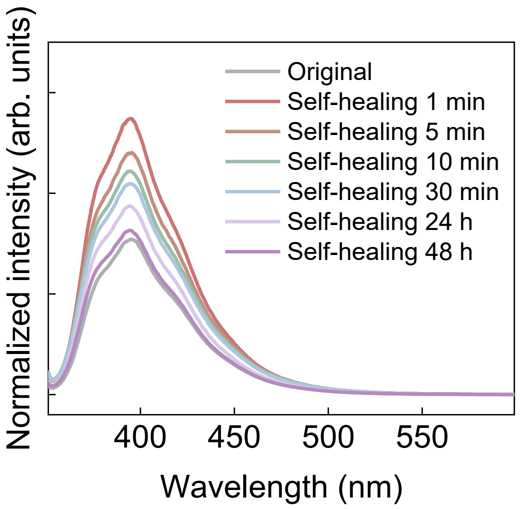

Supplementary Fig. 16. Fluorescence test of copolymer. The fluorescence intensity variation of P3 during self-healing process.

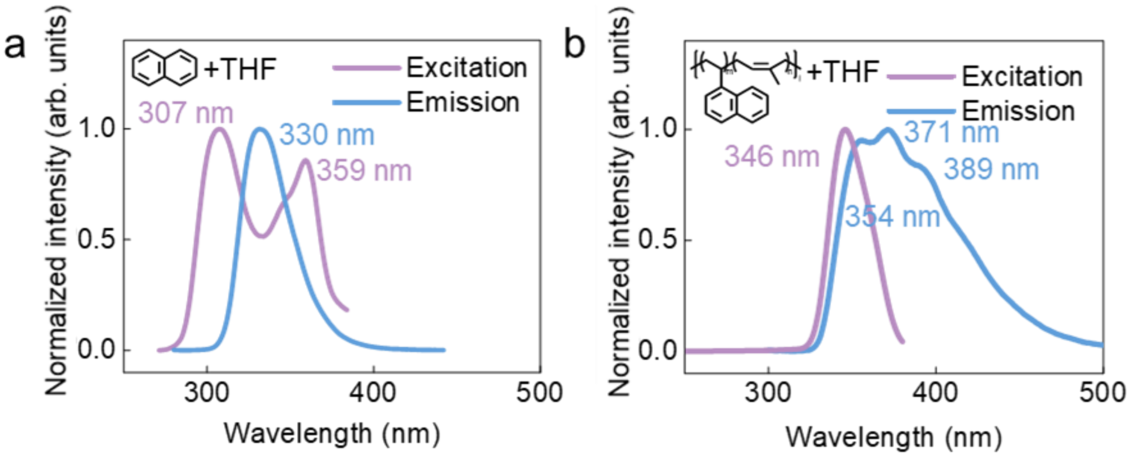

Supplementary Fig. 17. Fluorescent spectra of Naphthalene and PNI copolymer dissolved in THF. **a**) Naphthalene dissolved in THF (1 mM). **b**) P3 dispersed in THF (20 g/L).

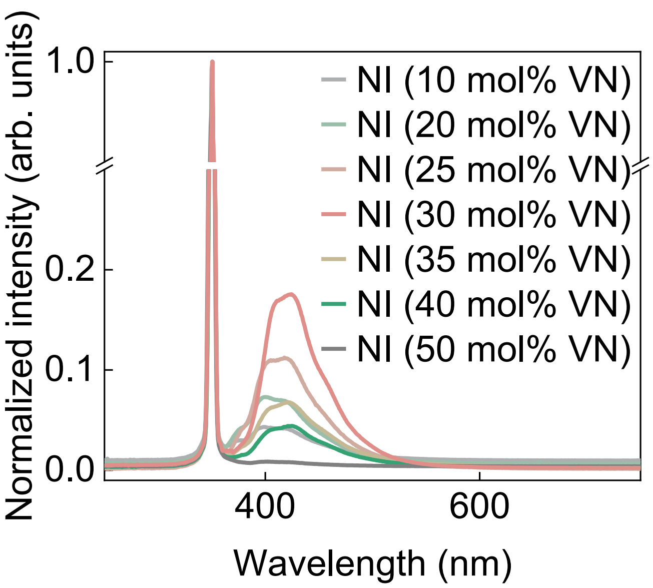

Supplementary Fig. 18. Fluorescence test of copolymer. Fluorescent emission spectra of PNI copolymer with different content of VN (10, 20, 25, 30, 35, 40, and 50 mol%).

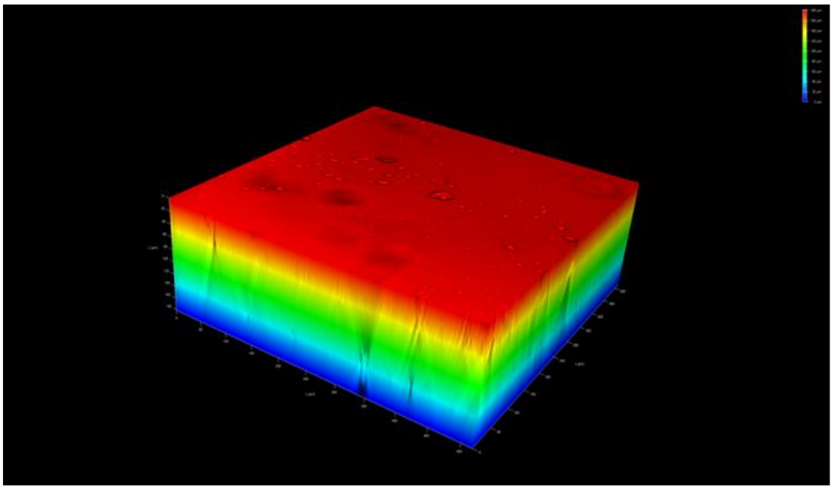

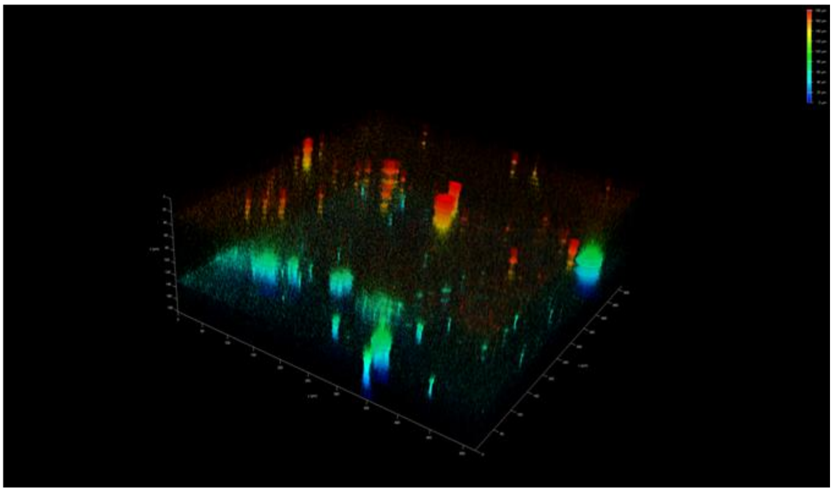

Supplementary Fig. 19. Fluorescent confocal test of copolymer. The 3D reconstruction of P3 block by Z-stack scan of the fluorescent confocal microscope.

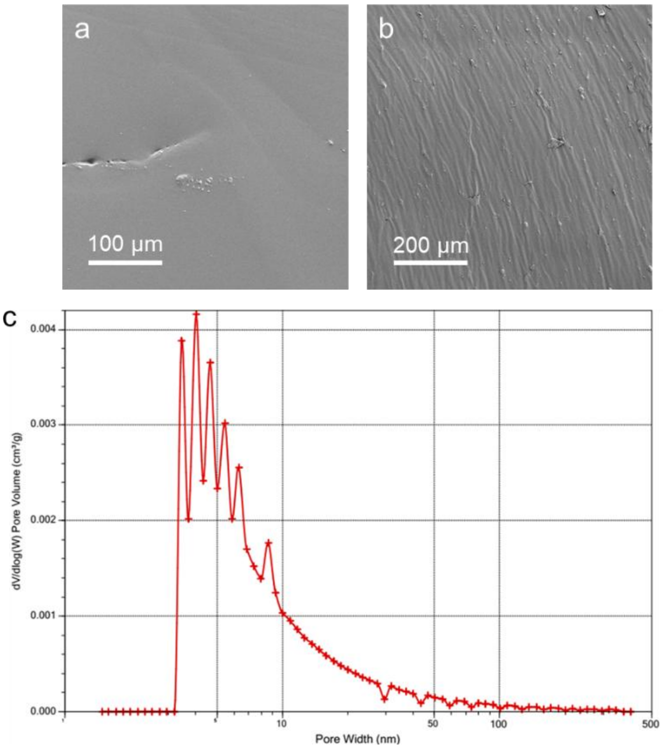

Supplementary Fig. 20. Scanning electron microscope (SEM) images and BET test result of P3. **a**) and **b**) Scanning electron microscope (SEM) images of P3. **c**) Pore size distribution curve of P3 gained by Brunauer-Emmett-Teller test (BET Surface Area: 0.4417 m^2/g).

Supplementary references

1. IUPAC, Compendium of chemical terminology, in: (the "Gold Book"). Compiled by A. D. McNaught and A. Wilkinson, second ed., Blackwell Scientific Publications, Oxford, 1997 https://doi.org/10.1351/goldbook. Online version (2019-) created by S. J. Chalk. ISBN 0-9678550-9-8.

2. J.-C. Bünzli, S.V. Eliseeva, Basics of lanthanide photophysics, springer series on fluorescence, in: P. Hänninen, H. Härmä (Eds.), Lanthanide Luminescence: Photophysical, Analytical and Biological Aspects, vol. 7,

Springer-Verlag Berlin Heidelberg, 2011, ISBN 978-3-642-21022-8 (Chapter 2).

3. Wong, K.-L., Bünzli, J.-C. G. & Tanner, P. A. Quantum yield and brightness. *J. Lumin.* **224**, 117256 (2020).

4. Fries, F.; Reineke, S., Statistical treatment of Photoluminescence Quantum Yield Measurements. *Sci. Rep.* **9** (1), 15638 (2019).
