# Supplementary material for: Polymer-constrained excimer enables flexible and self-healable optoelectronic elastomer for mechanical sensor
Source: Nat Commun. 2025 Nov 25;16:10500. doi: 10.1038/s41467-025-65539-9 (PMC12647883; doi:10.1038/s41467-025-65539-9)
Supplement: Supplementary file 2 — Description of Additional Supplementary Files [file 41467_2025_65539_MOESM2_ESM.pdf]

Description of Additional supplementary files

Supplementary movie 1: This movie demonstrates that the water bag prepared by P3 possesses the capability of rapid self-healing after being punctured.

Supplementary movie 2: This movie demonstrates that the film prepared by P3 exhibits rapid self-healing capability after being cut.

Supplementary movie 3: A movie showcasing the application of sensors constructed from P3 material on a robot hand: Different performance under natural light and ultraviolet light.
